# Supplementary material for: A bottom-up reward pathway mediated by somatostatin neurons in the medial septum complex underlying appetitive learning
Source: Nat Commun. 2022 Mar 7;13:1194. doi: 10.1038/s41467-022-28854-z (PMC8901785; doi:10.1038/s41467-022-28854-z)
Supplement: Supplementary file 1 — Supplementary Information [file 41467_2022_28854_MOESM1_ESM.pdf]

Supplementary Figures

A Bottom-up Reward Pathway Mediated by Somatostatin Neurons in the Medial Septum Complex Underlying Appetitive Learning

Li Shen[1, †], Guang-Wei Zhang[1, †], Can Tao[1], Michelle B. Seo[1,2], Nicole K. Zhang[1], Junxiang J. Huang[1,3], Li I. Zhang[1,4,*] & Huizhong W. Tao[1,4,*]

[1]Zilkha Neurogenetic Institute, Keck School of Medicine, University of Southern California, 1501 San Pablo Street, Los Angeles, California 90033, USA;

[2]Neuroscience Graduate Program, University of Southern California, Los Angeles, California 90089, USA;

[3]Graduate Programs in Biomedical and Biological Science, Keck School of Medicine, University of Southern California, Los Angeles, California 90033, USA;

[4]Department of Physiology & Neuroscience, Keck School of Medicine, University of Southern California, 1501 San Pablo Street, Los Angeles, CA 90033, USA.

[†] Authors contributed equally to this work.

[*] Correspondence should be addressed to: H.W.T (htao@usc.edu) or L.I.Z (liizhang@usc.edu)

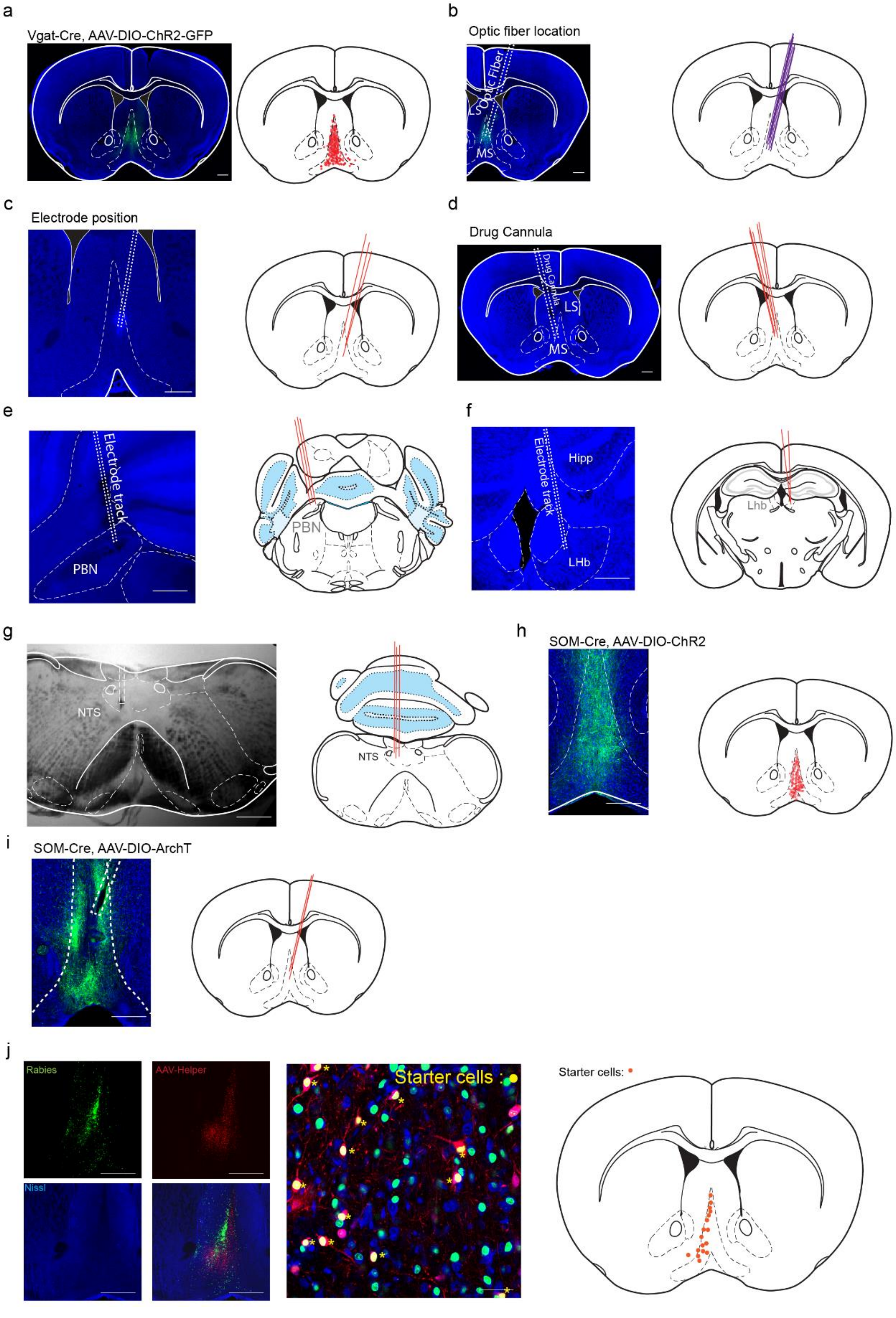

a
Vgat-Cre, AAV-DIO-ChR2-GFP
b
Optic fiber location
Optic Fiber
LS
MS
c
Electrode position
d
Drug Cannula
Drug Cannula
LS
MS
e
Electrode track
PBN
PBN
f
Electrode track
Hipp
LHb
Lhb
g
NTS
NTS
h
SOM-Cre, AAV-DIO-ChR2
i
SOM-Cre, AAV-DIO-ArchT
j
Rabies
AAV-Helper
Nissl
Starter cells :
Starter cells:

Supplementary Figure 1 | Post hoc histological verifications of viral expression and placements of electrodes and optic fibers.

a, An example coronal section showing the expression of ChR2-EYFP in a Vgat-Cre mouse. Right, superimposed ChR2-EYFP expressing cell locations across animals. Each small dot represents one cell (n = 7 mice). **b**, An example coronal section showing the track of the optic fiber implanted a Vgat-Cre mouse expressing ChR2-EYFP. Right, superimposed tracks of optic fibers across experiments (n = 7 mice). **c**, An example coronal section showing the track of the optrode electrode in MS. Right, superimposed tracks of electrodes (n = 3 mice). Each line represents one track. **d**, An example coronal section showing the track of drug cannula in MS. Right, superimposed tracks of cannulas (n = 5 mice). **e**, An example coronal section showing the track of the electrode in PBN. Right, superimposed tracks of electrodes (n = 3 mice). **f**, An example coronal section showing the track of electrode in LHb. Right, superimposed tracks of electrodes (n = 2 mice). **g**, An example bright field image showing the track of electrode in NTS. Right, superimposed tracks of electrodes (n = 3 mice). **h**, An example coronal section showing the expression of ChR2-EYFP in a SOM-Cre mouse. Right, superimposed ChR2-EYFP expressing cell locations (n = 3 mice). **i**, An example coronal section showing the track of optic fiber implanted in a SOM-Cre mouse with ChR2-EYFP expression in MS. Right, superimposed tracks of electrodes (n = 3 mice). **j**, Images of pseudo-yped rabies virus expression (n= 3 mice). Yellow stars indicate starter cells which were transduced by both AAV-helper and pseudo-typed rabies virus. Right, superimposed starter cell locations. Scale bar: 500 μm in all panels.

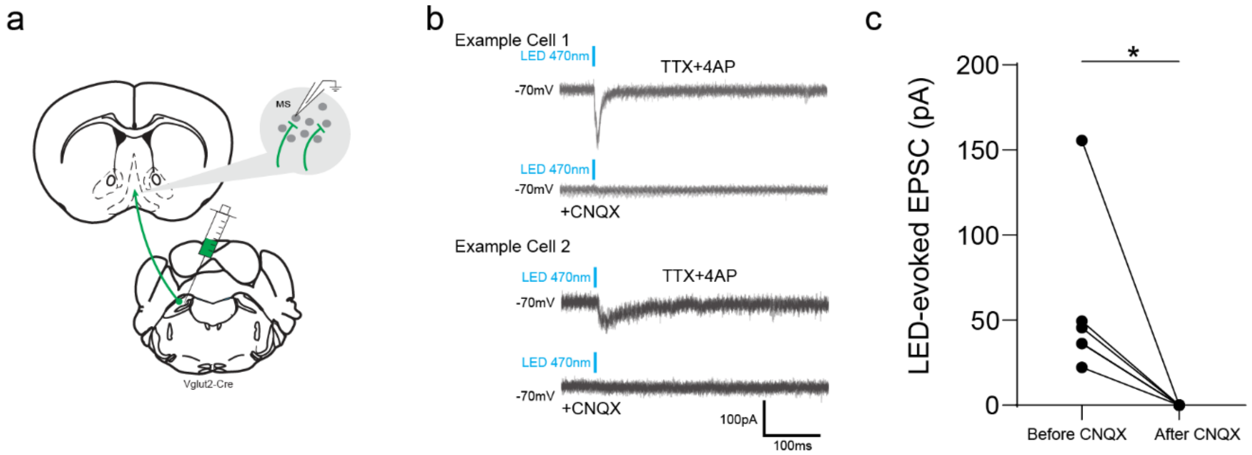

Supplementary Figure 2 | AMPA-receptor mediated synaptic currents of the excitatory PBN-MS projection.

a, Illustration of the experimental scheme. AAV-DIO-ChR2 was injected into PBN while in vitro brain slice recording was performed in MS. **b**, Two example neurons exhibited light-evoked inward synaptic currents recorded at -70mV in the presence of TTX and 4AP. Perfusion of CNQX completely blocked the currents. **c**, Amplitudes of light-evoked synaptic currents before and after CNQX application (n = 5). *, p = 0.0002, ratio paired t test, two-sided. Source data are provided as a Source Data file.

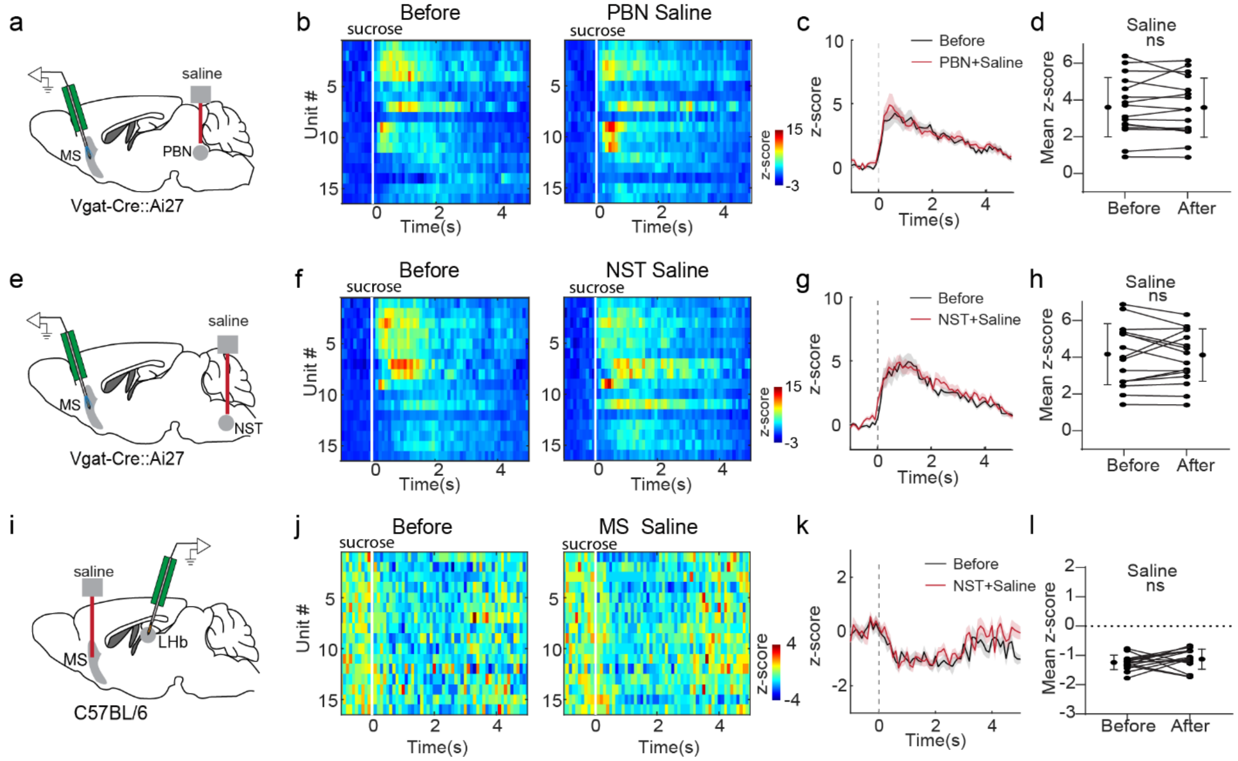

Supplementary Figure 3 | Control experiments for silencing PBN, NST and MS.

a, Optrode recording from MS GABAergic neurons while infusing PBN with saline vehicle for muscimol. **b**, Heatmap plot of Z-score for sucrose responses of MS GABAergic neurons (n = 16) before and after infusion of saline in PBN. **c**, Population average. All shades indicate s.e.m. **d**, Mean Z-score before and after infusing saline in PBN. Data points for the same neuron are connected with a line. "ns", not significant, p = 0.6147, paired t test, two-sided. **e-h**, Infusing saline in NST. "ns", not significant, p = 0.5867, paired t test, two-sided. **i-l**, Infusing saline in MS and recording from LHb neurons. "ns", not significant, p = 0.3455, paired t test, two-sided. Source data are provided as a Source Data file.

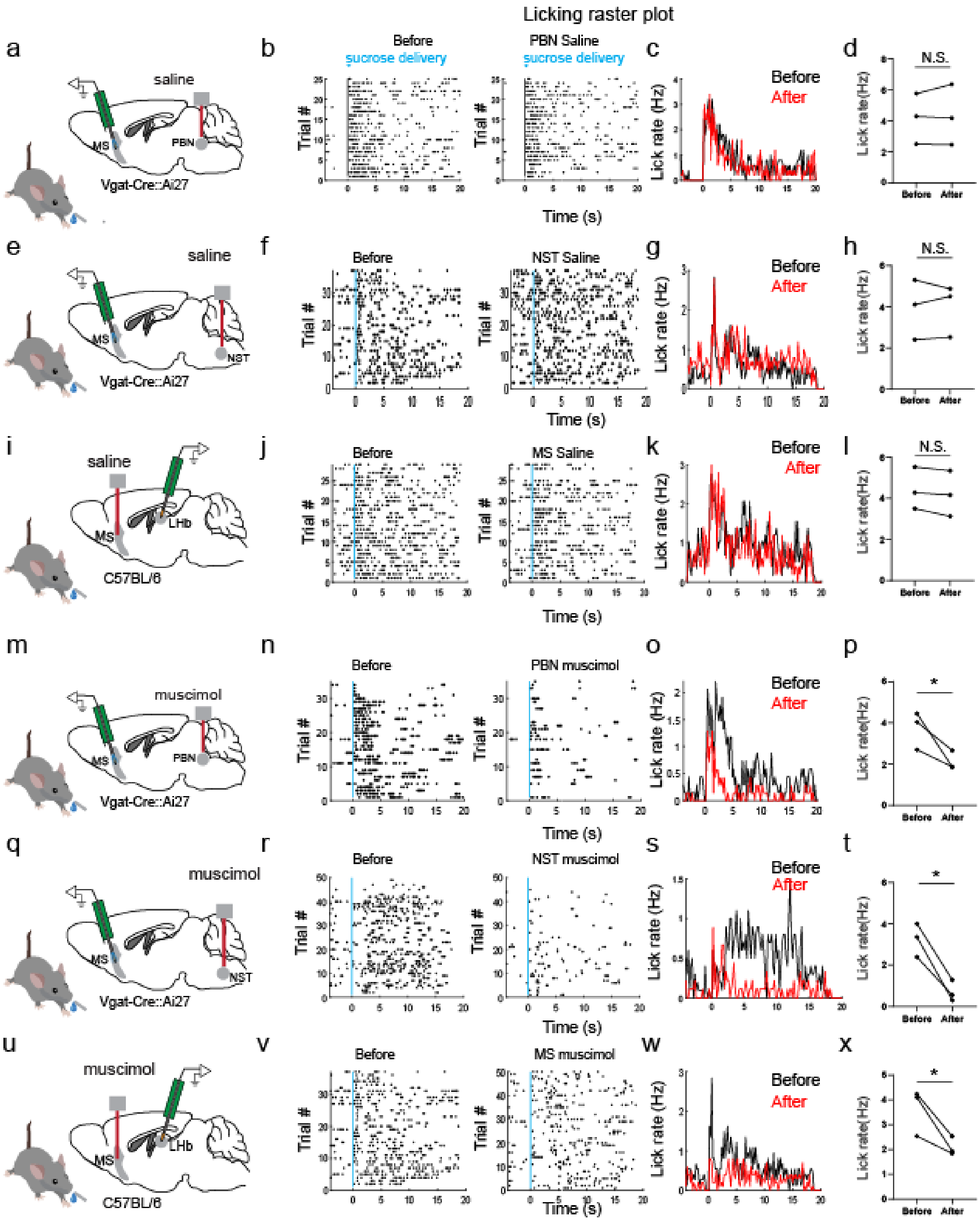

Supplementary Figure 4 | Sucrose licking behavior in control and silencing experiments.

a, Illustration of experimental scheme. Sucrose licking behavior was monitored when optrode recording was performed from MS GABAergic neurons. Saline was infused into PBN. **b**, Raster plot of licks to sucrose delivery before and after infusion of saline in PBN. **c**, Plot of lick rate before (in black) and after (in red) saline infusion. **d**, Mean lick rate before and after infusing saline in PBN. Data points for the same animal are connected with a line. N.S., p = 0.2209, not significant, paired t test, two-sided. **e-h**, Infusing

saline into NST, p=0.9204, paired t test, two-sided. **i-l**, Infusing saline into MS, p=0.103, paired t test, two-sided. **m-p**, Lick rate before and after infusing muscimol into PBN. *, p = 0.0479, paired t test, two-sided. **q-t**, Lick rate before and after infusing muscimol into NST. *, p = 0.0199, paired t test, two-sided. **u-x**, Lick rate before and after infusing muscimol into MS. *, p = 0.0476, paired t test, two-sided. Source data are provided as a Source Data file.

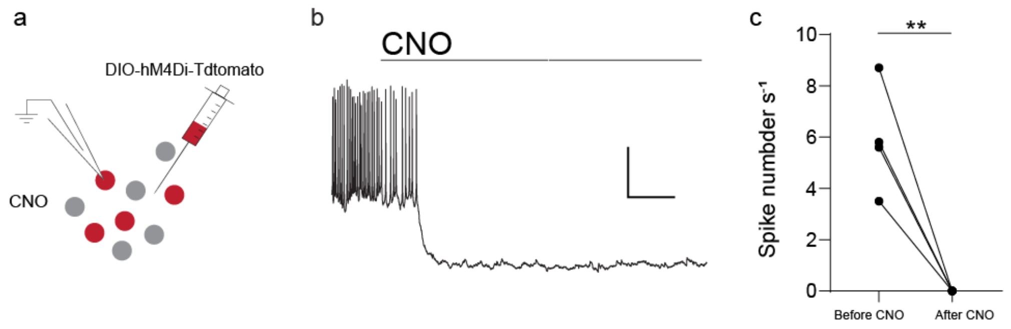

Supplementary Figure 5 | Efficiency of chemogenetic silencing of hM4Di-expressing neurons in MS.

a, Illustration of experimental scheme. Slice whole-cell recording was performed from neurons that expressed hM4Di (red). **b**, Example recorded trace showing that CNO application effectively suppressed spiking activity of an hM4Di-expressing MS neurons. Scale: 20mV, 5s. **c**, Mean spike rate (Hz) before and after CNO application. **, p= 0.0053, paired t test, one-sided. Source data are provided as a Source Data file.

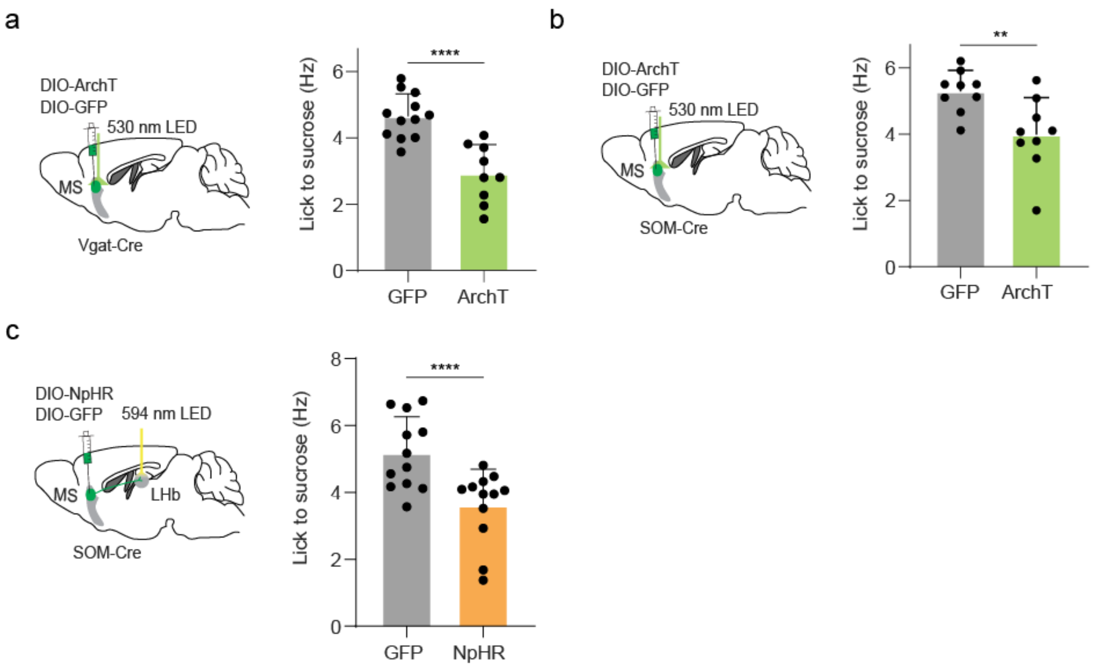

Supplementary Figure 6 | Lick rates to sucrose in optogenetic silencing experiments.

a, Left, illustration of experimental scheme. Either ArchT or GFP was expressed in MS GABAergic neurons in Vgat-Cre mice, with the optic fiber been implanted to target MS to deliver 530nm LED light. Right, lick rates to sucrose delivery in GFP or ArchT expressing mice. ****p<0.0001, t test, two-sided. **b**, Either ArchT or GFP was expressed in MS of SOM-Cre mice. Right, lick rates to sucrose in GFP or ArchT expressing mice. **p=0.0077, t test, two-sided. **c**, Either NpHR or GFP was expressed in MS of SOM-Cre mice, two-sided. Right, lick rates to sucrose in GFP or NpHR expressing mice. ****p=0.002, t test, two-sided. Source data are provided as a Source Data file.
