# Supplementary material for: A bottom-up reward pathway mediated by somatostatin neurons in the medial septum complex underlying appetitive learning
Source: Nat Commun. 2022 Mar 7;13:1194. doi: 10.1038/s41467-022-28854-z (PMC8901785; doi:10.1038/s41467-022-28854-z)
Supplement: Supplementary file 3 — Reporting Summary [file 41467_2022_28854_MOESM3_ESM.pdf]

nature portfolio

Corresponding author(s): Huizhong W. Tao and Li I. Zhang

Last updated by author(s): Feb 10, 2022

Reporting Summary

Nature Portfolio wishes to improve the reproducibility of the work that we publish. This form provides structure for consistency and transparency in reporting. For further information on Nature Portfolio policies, see our Editorial Policies and the Editorial Policy Checklist.

Statistics

For all statistical analyses, confirm that the following items are present in the figure legend, table legend, main text, or Methods section.

n/a	Confirmed	
☐	☒	The exact sample size (n) for each experimental group/condition, given as a discrete number and unit of measurement
☐	☒	A statement on whether measurements were taken from distinct samples or whether the same sample was measured repeatedly
☐	☒	The statistical test(s) used AND whether they are one- or two-sided<br>*Only common tests should be described solely by name; describe more complex techniques in the Methods section.*
☐	☒	A description of all covariates tested
☐	☒	A description of any assumptions or corrections, such as tests of normality and adjustment for multiple comparisons
☐	☒	A full description of the statistical parameters including central tendency (e.g. means) or other basic estimates (e.g. regression coefficient) AND variation (e.g. standard deviation) or associated estimates of uncertainty (e.g. confidence intervals)
☐	☒	For null hypothesis testing, the test statistic (e.g. F, t, r) with confidence intervals, effect sizes, degrees of freedom and P value noted<br>*Give P values as exact values whenever suitable.*
☒	☐	For Bayesian analysis, information on the choice of priors and Markov chain Monte Carlo settings
☒	☐	For hierarchical and complex designs, identification of the appropriate level for tests and full reporting of outcomes
☐	☒	Estimates of effect sizes (e.g. Cohen's d, Pearson's r), indicating how they were calculated

Our web collection on statistics for biologists contains articles on many of the points above.

Software and code

Policy information about availability of computer code

Data collection	We listed all software used in the experiments and for analysis in the Methods section. Customized Labview software (version 12.0.0) were used for in vivo electrophysiology and fiber photometry signal acquisition. In vitro brain slice electrophysiology signals were reordered using an Axopatch 700B amplifier (Molecular Devices). We used a customized Python software (version 3.6) for real-time animal detection and trigger optogenetic stimulation. Confocal images were captured using FluoView 1000 (Olympus, version FluoView 1000).
Data analysis	We listed all software used in the experiments and for analysis in the Methods section. We used customized Matlab code(Mathworks, version R2021a) and Offline Sorter (Plexon, version x64 v4.0) to analyze in vivo electrophysiology recording data. The version of T-Dist EM scan algorithm is version x64 v4.0 (Plexon). We used customized Python code (version 3.6) to track location and detect lick of animals (https://github.com/li-shen-amy/behavior). Customized Matlab (Mathworks, version R2021a) code were used to analyze the photometry signals. In vitro slice recording data were analyzed using pClamp (Molecular Devices, version 10.7.0) and customized Python codes (version 3.6). Prism version 8 software (GraphPad, version 8.0.2) and R (version 4.1.2) were used for statistical analysis.

For manuscripts utilizing custom algorithms or software that are central to the research but not yet described in published literature, software must be made available to editors and reviewers. We strongly encourage code deposition in a community repository (e.g. GitHub). See the Nature Portfolio guidelines for submitting code & software for further information.

Data

Policy information about availability of data

All manuscripts must include a data availability statement. This statement should provide the following information, where applicable:

- Accession codes, unique identifiers, or web links for publicly available datasets
- A description of any restrictions on data availability
- For clinical datasets or third party data, please ensure that the statement adheres to our policy

All relevant data are included in the manuscript, or detailed in the source data file. All other data that support the findings of this study are available from the corresponding authors upon request.

Data availability
The full datasets generated in the current study are available from the corresponding author upon reasonable request. Source data are provided with this paper.

Code availability
Animal detection and lick detection programs are available on https://github.com/li-shen-amy/behavior. DIO for the repository is 10.5281/zenodo.5992295.

Field-specific reporting

Please select the one below that is the best fit for your research. If you are not sure, read the appropriate sections before making your selection.

☒ Life sciences ☐ Behavioural & social sciences ☐ Ecological, evolutionary & environmental sciences

For a reference copy of the document with all sections, see nature.com/documents/nr-reporting-summary-flat.pdf

Life sciences study design

All studies must disclose on these points even when the disclosure is negative.

Sample size	For the single-cell in vivo recording, a prior power analysis was used to determine sample sizes. Otherwise, sample sizes were selected based on previous experiments from related research and literature.<br>1. Medial preoptic area antagonistically mediates stress-induced anxiety and parental behavior. Guang-Wei Zhang, Li Shen, Can Tao, A-Hyun Jung, Bo Peng, Zhong Li, Li I Zhang, Huizhong Whit Tao, Nature Neuroscience, DOI: 10.1038/s41593-020-00784-3<br>2. Dynamic salience processing in paraventricular thalamus gates associative learning. Yingjie Zhu, Gregory Nachtrab, Piper C Keyes, William E Allen, Liqun Luo, Xiaoke Chen. Science. DOI: 10.1126/science.aat0481<br>3. Functional circuit architecture underlying parental behaviour.Johannes Kohl, Benedicte M Babayan, Nimrod D Rubinstein, Anita E Autry, Brenda Marin-Rodriguez, Vikrant Kapoor, Kazunari Miyamishi, Larry S Zweifel, Liqun Luo, Naoshige Uchida, Catherine Dulac. Nature. DOI: 10.1038/s41586-018-0027-0
Data exclusions	No data were excluded.
Replication	Experimental findings were reliably reproduced among all subjects in all experiments comprised of multiple cohorts. In vivo recordings were conducted at least 3 cohorts of animals. Pharmacogenetic experiments were conducted with 6 cohorts of animals. Optogenetic experiments were conducted with at least 3 cohorts of animals. Slice recording were performed at least 2 cohorts of animals. Fiber photometry were performed at 2 cohorts of animals.
Randomization	Animals were randomly assigned to control and treatment groups. For the animals with multiple treatment, the sequence of treatment was randomized.
Blinding	Investigators were not blinded to group allocation or data collection, but the analyses of behavioral data were performed blind to the conditions of experiments as data obtained under different conditions were pooled together for an automatic batch analysis with computer software. For the other data, such as recording data, investigators were not blinded to group allocation or data collection, but the analyses of recorded data were performed blind to the conditions of experiments as data obtained under different conditions were pooled together for an semi-automatic batch analysis.

Reporting for specific materials, systems and methods

We require information from authors about some types of materials, experimental systems and methods used in many studies. Here, indicate whether each material, system or method listed is relevant to your study. If you are not sure if a list item applies to your research, read the appropriate section before selecting a response.

Materials & experimental systems

n/a	Involved in the study
☒	☐ Antibodies
☒	☐ Eukaryotic cell lines
☒	☐ Palaeontology and archaeology
☐	☒ Animals and other organisms
☒	☐ Human research participants
☒	☐ Clinical data
☒	☐ Dual use research of concern

Methods

n/a	Involved in the study
☒	☐ ChIP-seq
☒	☐ Flow cytometry
☒	☐ MRI-based neuroimaging

Animals and other organisms

Policy information about studies involving animals; ARRIVE guidelines recommended for reporting animal research

Laboratory animals	The Vglut2-ires-Cre (Jackson stock No. 016963), Vgat-ires-Cre (Jackson stock No. 016962), Som-ires-Cre (Jackson stock No. 013044), PV-ires-Cre (Jackson stock No. 008069), Ai14 (Cre-dependent tdTomato reporter line, Jackson stock No. 007914), Ai27 (Cre-dependent ChR2 reporter line, Jackson stock No. 012567) and C57BL/6 mice were obtained from the Jackson Laboratory. Experiments were performed in adult male and female mice (6-12 weeks old). All recordings and behavioral tests were conducted in the dark cycle. Mice were housed in a 12h light-dark cycle, with temperature range at 65-75F, and with 40%-60% humidity.
Wild animals	The study did not involve wild animals.
Field-collected samples	The study did not involve data collected from the field.
Ethics oversight	Animal experiments were conducted in accordance with the guidelines for the care and use of laboratory animals of US National Institutes of Health (NIH), and under protocols approved by institutional Animal Care and Use Committee at University of Southern California.

Note that full information on the approval of the study protocol must also be provided in the manuscript.
